# Associated factors, post infection child growth, and household cost of invasive enteritis among under 5 children in Bangladesh

**DOI:** 10.1038/s41598-021-92132-z

**Published:** 2021-06-17

**Authors:** Rina Das, Md. Ahshanul Haque, Mohammod Jobayer Chisti, A. S. G. Faruque, Tahmeed Ahmed

**Affiliations:** 1 Nutrition and Clinical Services Division, icddr,b, Dhaka, 1212 Bangladesh; 2 James P. Grant School of Public Health, BRAC University, Dhaka, 1212 Bangladesh; 3 Department of Global Health, University of Washington, Seattle, Washington, DC 98104 USA

**Keywords:** Microbiology, Diseases, Gastroenterology, Medical research, Risk factors

## Abstract

Both *Campylobacter-* and *Shigella*-induced invasive enteritis are common in under-5 Bangladeshi children. Our study aimed to determine the factors associated with *Campylobacter* and *Shigella* enteritis among under-5 children, the post-infection worsening growth, and the household cost of invasive enteritis. Data of children having *Shigella* (591/803) and *Campylobacter* (246/1148) isolated from the fecal specimen in Bangladesh were extracted from the Global Enteric Multicenter Study (GEMS) for the period December 2007 to March 2011. In multiple logistic regression analysis, fever was observed more frequently among shigellosis cases [adjusted OR 2.21; (95% CI 1.58, 3.09)]. Breastfeeding [aOR 0.55; (95% CI 0.37, 0.81)] was found to be protective against *Shigella.* The generalized estimating equations multivariable model identified a negative association between *Shigella* and weight-for-height z score [aOR − 0.11; (95% CI − 0.21, − 0.001)]; a positive association between symptomatic *Campylobacter* and weight-for-age *z* score [aOR 0.22; (95% CI 0.06, 0.37)] and weight-for-height z score [aOR 0.22; (95% CI 0.08, 0.37)]. Total costs incurred by households were more in shigellosis children than *Campylobacter*-induced enteritis ($4.27 vs. $3.49). Households with low-level maternal education tended to incur less cost in case of their shigellosis children. Our findings underscore the need for preventive strategies targeting *Shigella* infection, which could potentially reduce the disease burden, associated household costs, and child growth faltering.

## Introduction

*Campylobacter, Shigella, Salmonella,* and diarrheagenic *Escherichia coli* constitute the major bacterial pathogens that often cause acute invasive gastrointestinal infections^1–3^. A fundamental distinction among bacterial pathogens involves the capacity to invade intestinal epithelial cells and multiply within the gut mucosa, consequently exerting biological constraints on attempts to control their spread among susceptible populations^4^. *Shigella* continues to play a significant role in the etiology of dysentery and inflammatory diarrhea while *Campylobacter* is one of the most commonly isolated bacteria in infants with diarrhea. Watery or bloody diarrhea, fever, and abdominal pain are the characteristics of infection by *Shigella* and *Campylobacter*^5^. *Campylobacter*-induced acute gastroenteritis is difficult to differentiate from *Shigella*-associated gastroenteritis on the sole basis of clinical symptoms or routine stool examination and thus stool culture is required for a conclusive diagnosis of the causative organism.

Enteric infections can trigger inflammatory immune responses that can lead to chronic inflammation of the intestine and subsequent morphological changes. This interrupts nutrient absorption ability and leads to sequelae^6^. Studies investigating the effects of *Campylobacter* and *Shigella* on growth in children have been diverse and limited. The relationship between *Shigella* and enterotoxigenic *Escherichia coli*-associated with weight gain and linear growth has been investigated among children in Bangladesh aged between 0 and 5 years^7^. Household costs of diarrhea mediated by *Campylobacter* and *Shigella* have been reported to exert adverse consequences on the household economy^8^.

However, there is inadequate data on growth among children who are asymptomatic carriers or are suffering from invasive entireties by *Campylobacter* and *Shigella*. Moreover, there is an evident knowledge gap regarding the economic implications of infections caused by these two aforementioned infectious organisms in low- and- middle-income countries like Bangladesh.

Global Enteric Multicenter Study (GEMS) was a prospective case–control study conducted across 7 sites in sub-Saharan Africa and South Asia. In this present study, we aimed to compare the demographics, housing, animal exposure, clinical presentation, and associated factors among under-5 children with invasive enteritis associated with *Campylobacter* and *Shigella* infection in Bangladesh; evaluate the association between invasive enteritis and growth among under 5 children; and estimate household costs associated with invasive enteritis.

## Results

### Co-pathogens isolated from *Shigella* and *Campylobacter* positive children

In the Bangladesh site, *Shigella* and *Campylobacter* positive children having one or more bacterial, viral, and protozoal co-pathogens have been identified (Table S1). *Shigella flexneri* (28.77%) and *Shigella sonnei* (10.62%) were more frequently isolated among the cases with moderate-to-severe diarrhea (MSD) in comparison to the asymptomatic children. *Campylobacter jejuni* was found in almost 13% of both the symptomatic cases and asymptomatic healthy controls (Table S2).

### Characteristics of *Shigella*-positive children having moderate-to-severe diarrhea (MSD) in Bangladesh

About 44.3% of the *Shigella*-positive children with MSD were aged 24–59 months and half of them were female. *Giardia* was found to be the most common co-pathogen (Table 1) in the *Shigella* positive children compared to the *Shigella* negative children. A significantly higher proportion of under-five children with MSD and associated *Shigella* infection were stunted, wasted and underweight. The duration of diarrhea before coming to the facility was less. The children had more frequent visible blood in the stool, less vomiting, and more often presented with a history of fever during admission. Regarding the inclusion criteria for MSD, the children more commonly had dysentery and required hospital admission. Caregivers of the *Shigella*-positive children practiced handwashing less frequently before nursing the child/preparing baby food and after cleaning the child; one-fourth of them belonged to middle and upper middle-class families. *Shigella*-positive children were less often breastfed. Their stool examinations reported the habitual presence of fecal red blood cell (RBC) and mucus more frequently in comparison to the stool specimens of *Shigella*-negative children.

**Table 1:** Baseline characteristics of the *Shigella*-positive and *Campylobacter*-positive under 5 Bangladeshi children having MSD.

Characteristics	*Shigella*			*Campylobacter*		
	*Shigella*-positive n = 591 (%)	*Shigella*-negative n = 803 (%)	*p *value*	*Campylobacter*-positive n = 246 (%)	*Campylobacter*-negative n = 1148 (%)	*p *value*
**Age group (m)**						
0–11	72 (12.2)	478 (59.5)	–	161 (65.5)	389 (33.9)	–
12–23	257 (43.5)	219 (27.3)	< 0.001	70 (28.5)	406 (35.4)	< 0.001
24–59	262 (44.3)	56 (23.6)	< 0.001	15 (6.1)	353 (30.8)	< 0.001
**Gender (female)**	246 (41.6)	334 (41.6)	0.991	104 (42.3)	476 (41.5)	0.814
**Anthropometry**						
Wasted	151 (25.6)	144 (17.9)	0.001	30 (12.2)	265 (23.1)	< 0.001
Stunted	154 (26.1)	181 (22.5)	0.129	48 (19.5)	287 (25.0)	0.068
Underweight	219 (37.1)	234 (29.1)	0.002	52 (21.1)	401 (34.9)	< 0.001
MUAC (mean ± SD)	14.3 ± 1.1	14.3 ± 1.3	0.909	14.06 ± 1.2	14.3 ± 1.3	< 0.001
**Clinical features**						
History of duration of diarrhea (mean ± SD)	2.97 ± 1.56	3.07 ± 1.46	0.224	3.01 ± 1.44	3.03 ± 1.52	0.837
Visible blood in stool	520 (87.9)	518 (64.5)	< 0.001	212 (86.2)	826 (71.9)	< 0.001
Vomiting ≥ 3 times/ day	108 (18.3)	264 (32.9)	< 0.001	46 (18.7)	326 (28.4)	0.002
Fever on admission	505 (85.5)	557 (69.4)	< 0.001	159 (64.6)	903 (78.7)	< 0.001
**Indicators for MSD**						
Sunken eyes	29 (4.9)	191 (23.8)	< 0.001	21 (8.5)	199 (17.3)	0.001
Loss of skin turgor	7 (1.2)	54 (6.7)	< 0.001	6 (2.4)	55 (4.8)	0.108
IV rehydration needed	33 (5.6)	132 (16.4)	< 0.001	13 (5.3)	152 (13.2)	0.001
Dysentery	512 (86.6)	517 (64.4)	< 0.001	211 (85.8)	818 (71.3)	< 0.001
Required hospital admission	135 (22.8)	148 (18.4)	0.043	19 (7.7)	264 (23.0)	< 0.001
**Socio-demographic features**						
Primary caretaker (mother)	584 (98.8)	799 (99.5)	0.165	244 (99.2)	1139 (99.2)	0.963
**Mother’s education**						
Illiterate	59 (9.9)	98 (12.2)	0.196	27 (10.9)	130 (11.3)	0.875
**Household characteristics**						
Number of people live in house	5.9 ± 2.7	5.8 ± 2.8	0.457	6.3 ± 3.3	5.7 ± 2.6	0.008
Number of people sleep in house	5.9 ± 2.7	5.8 ± 2.8	0.471	6.2 ± 3.3	5.7 ± 2.6	0.009
Number of under-5 children at house	1.3 ± 0.6	1.4 ± 0.7	0.619	1.5 ± 0.8	1.3 ± 0.6	0.004
Predominant floor (earth/sand)	498 (84.3)	661 (82.3)	0.337	200 (81.3)	959 (83.5)	0.396
**Animal at house**						
Cow	301 (50.9)	391 (48.7)	0.409	130 (52.9)	562 (48.9)	0.268
Dog	468 (79.2)	657 (81.8)	0.219	207 (84.2)	918 (79.9)	0.113
Cat	479 (81.1)	680 (84.7)	0.074	213 (86.6)	946 (82.4)	0.113
Rodent/fowl	384 (64.9)	501 (62.4)	0.322	166 (67.5)	719 (62.6)	0.152
Goat	67 (11.3)	82 (10.2)	0.502	30 (12.2)	119 (10.4)	0.400
**Main source of drinking water**						
Tube well water	589 (99.7)	798 (99.4)	0.465	244 (99.2)	1143 (99.6)	0.455
Treat drinking water	29 (4.9)	32 (3.9)	0.406	9 (3.7)	52 (4.5)	0.545
**Fecal disposal**						
Toilet facility available	551 (93.2)	758 (94.4)	0.370	237 (96.3)	1072 (93.4)	0.083
**Hand washing practice**						
Before nursing/preparing baby food	144 (24.4)	223 (27.8)	0.154	73 (29.7)	294 (25.6)	0.189
After handling animals	176 (29.8)	235 (29.3)	0.835	74 (30.1)	337 (29.4)	0.821
After cleaning a child	227 (38.4)	344 (42.8)	0.097	99 (24.4)	472 (41.1)	0.801
**Hand wash material**						
Water and soap	74 (12.5)	98 (12.2)	0.859	24 (9.8)	148 (12.9)	0.176
**Wealth index**						
Poor	114 (19.3)	176 (21.9)	–	50 (20.3)	240 (20.9)	–
Lower middle	109 (18.4)	162 (20.2)	0.826	46 (18.7)	225 (19.6)	0.933
Middle	130 (22.0)	149 (18.6)	0.080	44 (17.9)	235 (20.5)	0.637
Upper middle	132 (22.3)	155 (19.3)	0.105	46 (18.7)	241 (20.9)	0.696
Rich	106 (17.9)	161 (20.1)	0.925	60 (24.4)	207 (18.0)	0.122
**Breastfeeding status**						
Breastfed	454 (76.8)	723 (90.0)	< 0.001	230 (93.5)	947 (82.5)	< 0.001
**Stool examination**						
RBC present in stool	302 (51.1)	235 (29.3)	< 0.001	107 (43.5)	430 (37.5)	0.078
Mucus present in stool	569 (96.3)	656 (81.7)	< 0.001	228 (92.7)	997 (86.9)	0.012
**Co-pathogens isolated in stool**						
*Giardia*	65 (11.0)	41(5.1)	< 0.001	22 (8.9)	84 (7.3)	0.383
*Cryptosporidium*	35 (5.9)	63 (7.9)	0.166	14 (5.7)	84 (7.3)	0.367
*Entamoeba histolytica*	43 (7.3)	50 (6.2)	0.438	18 (7.3)	75 (6.5)	0.655
EAEC	121 (20.5)	212 (26.4)	0.010	67 (27.2)	266 (23.2)	0.175
**Outcome**						
Duration of hospital stay (mean ± SD)	4.1 ± 1.9	4.4 ± 1.9	0.042	4.8 ± 2.0	4.3 ± 1.9	0.072

Stunting: HAZ <  − 2, (%; for < 5 years of age), Underweight: WAZ <  − 2, (%; for < 5 years of age), Wasted: WHZ <  − 2, (%; for < 5 years of age), MUAC, (mean; for < 5 years of age) Mid upper arm circumference, RBC, red blood cell, Diarrhea: 3 or more loose watery stool/day, Fever: measured at least 38°C or parental perception; WASH, water, sanitation, and hygiene; EAEC, enteroaggregative *E. coli*; SD, standard deviation.

*Variable was added in multiple models if *p* < 0.25 in bi-variate model.

### Characteristics of *Campylobacter*-positive children having MSD

About 65% of the *Campylobacter*-positive children with MSD were aged 0–11 months and 42% of them were female; less often had malnutrition; mostly presented with visible blood in stool and fever during admission, less frequently presented with vomiting. Among the inclusion criteria of MSD, more children had dysentery and required less hospital admission. They had a large family size, and more under-5 children in the house; more frequently had toilet facility at the house; practiced handwashing more commonly before nursing the child/preparing baby food; less often washed hand with soap. Eighteen percent of children were from wealthy families and were more commonly breastfed. Their stool examinations reported the frequent presence of fecal RBC and mucus compared to the stool specimens of *Campylobacter*-negative MSD children. EAEC was found more often as co-pathogens (Table 1) in the *Campylobacter*-positive children.

Multiple logistic regression reveals that MSD children with *Shigella* were significantly associated with the presence of blood in stool [OR 2.41 (95% CI 1.56, 3.70)], usually presented with fever [OR 2.21 (95% CI 1.58, 3.09)], less often had features of dehydration like sunken eyes [OR 0.31 (95% CI 0.18, 0.52)]; and were less frequently breastfed [OR 0.55 (95% CI 0.37, 0.81)]. However, fecal RBC [OR 1.61 (95% CI 1.20, 2.17)] and mucus [OR 2.46 (95% CI 1.38, 4.37)] were more often reported during stool examinations among MSD children with *Shigella* (Table 2) compared to the children without fecal *Shigella*. Conversely, *Campylobacter* positive MSD children were associated with blood in stool [OR 2.57 (95% CI 1.72, 3.81)]; more often sought healthcare service; and had a significantly fewer history of fever [OR 0.70 (95% CI 0.51, 0.96)] upon attendance to the health facility compared to the children without fecal *Campylobacter* (Table 2).

**Table 2:** Results of multiple logistic regression after exploring the associated factors of *Shigella* and *Campylobacter* infections in MSD children aged less than 5 years in Bangladesh.

Factors	*Shigella*		*Campylobacter*	
	aOR (95% CI)	*p *value	aOR (95% CI)	*p *value
**Age group (months)**				
0–11	Reference		Reference	
12–23	8.88 (6.37–12.37)	< 0.001	0.44 (0.32–0.61)	< 0.001
24–59	16.84 (11.54–25.59)	< 0.001	0.10 (0.06–0.18)	< 0.001
**Gender**				
Male	Reference		Reference	
Female	0.88 (0.67–1.16)	0.358	1.08 (0.80–1.44)	0.626
**Clinical feature**				
Blood in stool				
No	Reference		Reference	
Yes	2.41 (1.56–3.70)	< 0.001	2.57 (1.72–3.81)	< 0.001
Fever				
No	Reference		Reference	
Yes	2.21 (1.58–3.09)	< 0.001	0.70 (0.51–0.96)	0.027
Sunken eyes				
No	Reference			
Yes	0.31 (0.18–0.52)	< 0.001	–	
**Breastfeeding status**				
Non-breastfed	Reference			
Breastfed	0.55 (0.37–0.81)	0.003	–	
**Laboratory findings**				
RBC in stool				
No	Reference			
Yes	1.61 (1.20–2.17)	0.002	–	
Mucus in stool				
No	Reference			
Yes	2.46 (1.38–4.37)	0.002	–	

Fever: measured at least 38 °C or parental perception, RBC, red blood cell; aOR, adjusted odds ratio; CI, confidence interval.

### Child growth with fecal *Shigella* and *Campylobacter*

**Figure 1:**
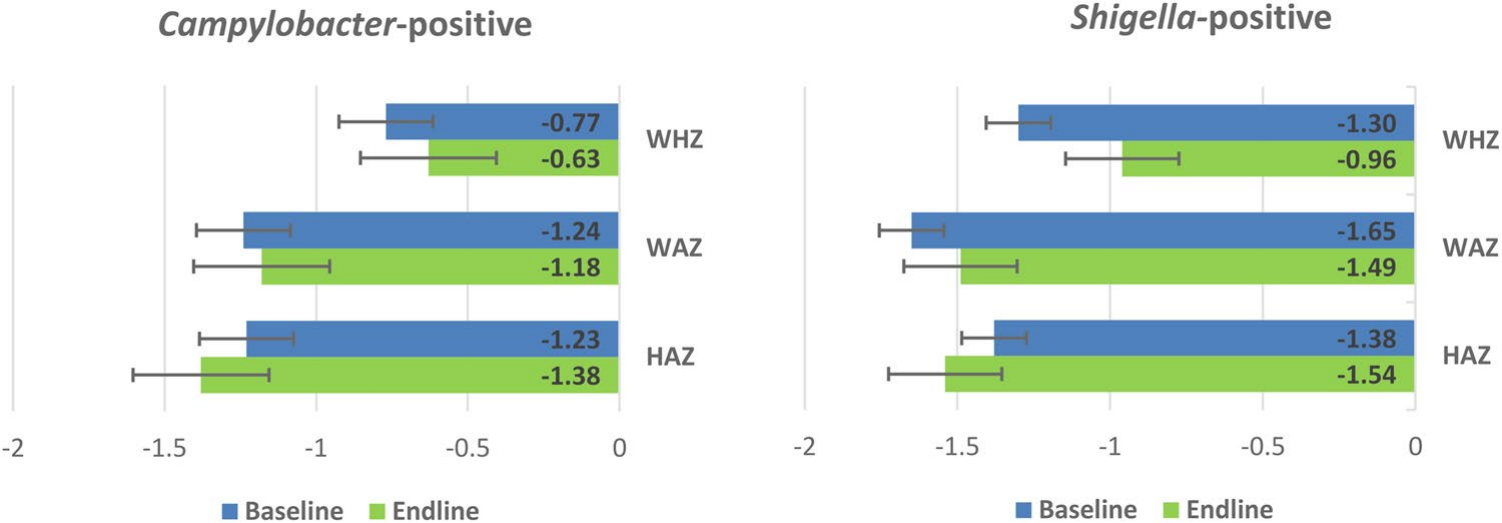
Mean baseline and endline height-for-age z score (HAZ), weight-for-age z score (WAZ), and weight-for-height z score (WHZ) among the fecal *Shigella*-positive and *Campylobacter*-positive under 5 children from Bangladesh.

In the unadjusted model, the mean height-for-age z score (HAZ) was found to be elevated at the endline but the mean weight-for-age z score (WAZ) and weight-for-height z score (WHZ) were reduced in the endline (Fig. 1) among the fecal *Shigella* and *Campylobacter*-positive under-5 children.

In Table 3, the findings of generalized estimating equations (GEE) modeling have been presented. A significant association between *Shigella* infection and WHZ was shown in the unadjusted GEE model. After adjusting for other covariates, namely: age, gender, MSD, breastfeeding status, mother’s education, number of people regularly sleeping in the house, number of under-5 children at house, handwashing material, hand washing before nursing a child and after cleaning the child, access to toilet facility, the main source of drinking water, wealth index, co-pathogens *Cryptosporidium* and *Giardia,* comorbidity (pneumonia, diarrhea, dysentery, malaria, typhoid), the multivariable model revealed a significant negative association between WHZ [Coefficient: − 0.11 (95% CI − 0.21, − 0.001) and *Shigella*, indicating that children having *Shigella* infection irrespective of MSD had poor growth. On the other hand, a significant positive association of WAZ [Coef.: 0.22 (95% CI 0.06, 0.37)] and WHZ [Coef.: 0.22 (95% CI 0.08, 0.37)] with *Campylobacter* infections indicated symptomatic children having *Campylobacter* infection had improved growth (Table 3). The analysis was replicated without adjusting the significant co-pathogens (*Cryptosporidium* and *Giardia*) and no difference in the adjusted mean growth was found (Table S3).

**Table 3:** Association of fecal *Shigella and Campylobacter* with a child’s HAZ, WAZ, and WHZ: results of generalized estimating equations modeling (dependent variable—HAZ, WAZ, and WHZ).

Pathogens	*Z* score	Unadjusted		Adjusted^a^		Adjusted^a^			
		All		All		Asymptomatic		Symptomatic	
		Coef. (95% CI)	*p* value	Coef. (95% CI)	*p *value	Coef. (95% CI)	*p *value	Coef. (95% CI)	*p *value
*Campylobacter*-positive	HAZ	0.06 (− 0.03, 0.15)	0.221	0.02 (− 0.07, 0.10)	0.732	− 0.02 (− 0.14, 0.09)	0.677	0.09 (− 0.07, 0.24)	0.268
	WAZ	0.11 (0.02, 0.20)	0.021	0.05 (− 0.04, 0.14)	0.257	− 0.04 (− 0.15, 0.08)	0.530	0.22 (0.06, 0.37)	**0.005**
	WHZ	0.10 (0.01, 0.19)	0.023	0.04 (− 0.05, 0.12)	0.397	− 0.07 (− 0.17, 0.04)	0.240	0.22 (0.08, 0.37)	**0.003**
*Shigella*-positive	HAZ	− 0.11 (− 0.21, − 0.02)	0.015	0.001 (− 0.11, 0.11)	0.972	− 0.07 (− 0.34, 0.20)	0.597	0.03 (0.09, 0.16)	0.644
	WAZ	− 0.31 (− 0.40, − 0.21)	< 0.001	− 0.06 (− 0.17, 0.05)	0.282	− 0.04 (− 0.32, 0.24)	0.784	− 0.04 (− 0.17, 0.09)	0.511
	WHZ	− 0.41 (− 0.50, − 0.32)	< 0.001	− 0.11 (− 0.21, − 0.001)	**0.046**	− 0.01 (− 0.28, 0.26)	0.939	− 0.10 (− 0.22, 0.02)	0.110

Coef., coefficient; CI, confidence interval; HAZ, height-for-age z score; WAZ, weight-for-age z score; and WHZ, weight-for-height z score.

^a^Adjusted for age, gender, MSD, breastfeeding status, mother’s education, number of people regularly sleep in the house, number of under-5 children at house, hand washing before nursing a child and after cleaning the child, handwashing material, main source of drinking water, available toilet facility, wealth index, copathogen: *Cryptosporidium* and *Giardia*, and comorbidity (malaria, typhoid, pneumonia, diarrhea, and dysentery).

### Expenditures by type of cost

Among cases with nonzero costs (*Shigella*, n = 591, and *Campylobacter*, n = 246), the total cost was higher in the *Shigella*-positive cases ($4.17) compared to the *Campylobacter*-positive cases ($3.49). Mean direct medical costs to households were similar in both *Shigella* and *Campylobacter* ($2.95 vs $2.32). The indirect cost was 3.22 USD among the *Shigella*-mediated enteritis cases and 4.74 USD for *Campylobacter*-mediated enteritis cases, but the total direct cost was similar in both the *Shigella*-mediated and *Campylobacter*-mediated enteritis cases (Table S4).

### Determinants of costs

The relationship between the wealth index, gender, education, age, severity and duration, household direct medical costs and overall costs were investigated (Table 4). There was no difference in total costs for both the *Shigella*-mediated and *Campylobacter*-induced enteritis. Both enteritis indicated a higher cost with higher levels of maternal education, especially for household total costs in the case of *Shigella*-mediated enteritis (*p* = 0.046). Higher household total costs with a greater duration of hospital stay were observed in both cases (*p* < 0.05) (Table 4).

**Table 4:** Household direct medical costs and total household costs for *Shigella* and *Campylobacter* MSD by socioeconomic, demographic, and illness characteristics in Bangladesh.

Variables	*Shigella*-positive				*Campylobacter*-positive			
	Direct medical cost		Total household cost		Direct medical cost		Total household cost	
	Mean cost	ANOVA^a^	Mean cost	ANOVA^a^	Mean cost	ANOVA^a^	Mean cost	ANOVA^a^
	USD		USD		USD		USD	
**Wealth index**								
Poor	3.04	*p* = 0.968	4.43	*p* = 0.730	2.40	*p* = 0.298	3.98	*p* = 0.376
Lower middle	2.85		3.94		2.42		3.48	
Middle	2.98		4.37		2.52		3.32	
Upper middle	2.90		3.93		2.15		4.18	
Rich	2.97		4.21		2.18		2.70	
**Sex**								
Male	2.85	*p* = 0.182	4.11	*p* = 0.614	2.23	*p* = 0.080	3.37	*p* = 0.601
Female	3.08		4.26		2.45		3.66	
**Mother’s education**								
Literate	3.00	*p* = 0.049	4.27	*p* = 0.046	2.33	*p* = 0.745	3.45	*p* = 0.609
Illiterate	2.45		3.28		2.26		3.88	
**Age group (months)**								
0–11	2.70	*p* = 0.037	4.01	*p* = 0.080	2.31	*p* = 0.657	3.43	*p* = 0.769
12–23	2.77		3.84		2.30		3.74	
24–59	3.18		4.55		2.55		2.97	
**Duration of hospital stay (days)**								
1–3	3.28	*p* = 0.004	5.30	*p* < 0.001	3.31	*p* = 0.608	5.35	*p* = 0.045
≥ 4	4.79		8.95		53.59		9.65	

^a^ANOVA; Analysis of variance; USD: US Dollar; *t *test was conducted when categories were two.

## Discussion

In our study, children with *Shigellosis* and *Campylobacter* infections presenting with dysentery were compared to fecal *Shigella* and *Campylobacter* negative children. Our findings were comparable to other studies^9–11^, despite being unable to exclude the co-pathogens responsible for dysentery in shigellosis and *Campylobacter* infections.

*Campylobacter-*positive children reported a significantly lower incidence of fever at admission in comparison to *Campylobacter-*negative children. However, fever was more common in the case of *Shigella*-positive children compared to *Shigella*-negative children. It was probably due to mild, often self-limiting *Campylobacter* infections that needed only supportive treatment^12^. This observation was similar to a study conducted in the north of Israel^12^. Henceforth, fever on admission associated with dysentery will be helpful for the clinicians to differentiate between shigellosis and *Campylobacter* infections among under-5 children.

In our study, *Shigella-*positive children less often presented with sunken eyes. Findings from a study conducted in a large urban diarrhea treatment facility in Bangladesh reported frequent presentation of shigellosis with some or severe dehydration in children^13–15^. In the case of *Campylobacter* infection, we observed no association with inclusion criteria of MSD and our findings were consistent with a study among Canadian children, where dehydration was not reported to be a common presenting feature of *Campylobacter*-mediated enteritis^16^ and a similar finding was observed in a large waterborne outbreak of *Campylobacter jejuni* in Norway^17^.

In our analysis, breastfeeding was found to be a protective factor for shigellosis. Very little is known about the impact of breastfeeding on *Shigella*-related diarrheal diseases. Another research from Bangladesh studied the children up to the first three years of age and found that breastfed children up to 35 months of age had a higher level of immunity against severe shigellosis^18^. In rural Mozambique, breastfeeding was also found protective for diarrhea caused by *Shigella*^19^.

We observed a significant negative association of *Shigella*-mediated enteritis with weight-for-height *z* score. In other studies, researchers have indicated a similar type of results^7,20^. Malnourished children have been found to present with longer duration of illness and deep ulcerations in the colon. Both acute and prolonged episodes of shigellosis may result in extensive loss of blood from the colonic ulcerations^21^. Thus, in addition to the other effects of diarrhea, shigellosis results in loss of serum protein that, for children on a marginal diet, must be compensated by increased protein intake for optimal growth to occur^22,23^. This fecal protein loss may have been partially responsible for growth faltering in children with shigellosis. However, in our study, we found no effect of shigellosis in limiting linear growth.

In several studies, researchers observed an association between *Campylobacter* infection and reduced weight gain as well as reduced linear growth^20,24^. However, in our study, we did not find any association of asymptomatic *Campylobacter* with child anthropometry indices. There was a positive association between symptomatic *Campylobacter* infection with child growth. This may be because of the treatment of symptomatic episodes with antibiotics. Thus, antibiotic treatment may be a confounding factor in estimating the true effect of Campylobacteriosis on child growth. Similar findings were observed in a systematic review and meta-analysis of *Campylobacter* infection^25^. In our study, stunting, wasting, or underweight, in any form, were not associated with invasive enteritis caused by *Campylobacter* or *Shigella*. Since the risk of diarrheal disease among severely malnourished children may be higher compared to that in the well-nourished children, our population may be less than ideally suited to disentangling this impact, limiting our ability to assess whether the association between *Campylobacter* and growth during enrollment was mediated by the nutritional deficit.

In our study population, *Cryptosporidium* and *Giardia* were prevalent and they are known to influence the growth of children^26–28^. However, their impact can also be eliminated in this situation, as we controlled their effect during GEE modeling for both *Campylobacter* and *Shigella* infections. The analysis was repeated to those children not infected with *Cryptosporidium* and *Giardia.* The difference in adjusted mean growth was measured in terms of the HAZ, WAZ, and WHZ score, which was observed to be almost the same. Other studies which used a single assessment of nutritional status to establish a possible link between *Shigella* or *Campylobacter* with malnutrition have not been able to distinguish between the growth effect of *Shigella* or *Campylobacter*-mediated enteritis or the increased vulnerability of malnourished children to infection.

Medical costs differed by sex, with direct costs being higher for girls suffering from *Shigella* enteritis, and higher for boys suffering from *Campylobacter* enteritis, with no difference in overall costs between *Shigella* and *Campylobacter*-mediated enteritis. Given the evidence revealed in the literature that household spending on health care, food, and education sometimes favors boys over girls, these findings warrant further exploration^29,30^. We also observed evidence of lower total costs for children with lower maternal education levels in the case of *Shigella*-mediated enteritis. Literate mothers incurred higher costs for the treatment of *Shigella* infections in comparison to illiterate mothers. This was more likely because of illiteracy to curtail or prolong care-seeking. This brings with it the danger that delayed care leading to more adverse outcomes among illiterate mothers’ children. We do not have any ready explanation for this observation but further studies may address these issues.

Total medical costs were amplified by the increased duration of hospital stay in cases of both *Campylobacte*r and *Shigella*-mediated enteritis. Another study from Northern Ghana also reported higher hospital costs in inpatients than those who received outpatient treatment^31^.

Unbiased sampling following a standard protocol^32^, a large sample size^33^, and high-quality laboratory performance^32^ were the strengths of our analysis. In this study, we aimed to determine the factors associated with both symptomatic and asymptomatic *Shigella* and *Campylobacter* infections among under-5 children. A single home follow-up visit approximately 60 days after enrollment was a valuable addition to the results of this research, which allowed us to understand the growth outcomes of children during the vulnerable times of their lives.

Nevertheless, our study has several limitations warranting a careful interpretation of the results when explaining these findings. It includes the inability to determine the relationship between maternal age and BMI, gestational age, and birth weight data for child growth failure. Due to a limited number of samples, we could not conclude the differential effects of *Campylobacter* and *Shigella* by species. This study did not evaluate the antimicrobial susceptibility patterns. Additionally, the cost of adverse outcomes and mental effects (such as distress and tiredness) of diarrheal disease caregivers have not been clarified in the current study. Moreover, the study was conducted in a sub-district of Bangladesh, so the results may not be generalizable for the whole country.

In conclusion, the use of clinical predictors may make it possible to target appropriate empiric antimicrobial therapy for children most likely to have invasive enteritis in resource-constrained settings. Our findings underscore the need for preventive strategies targeting *Shigella*, which could potentially reduce the disease burden and its sequelae such as child growth faltering during the first 5 years of life. Results also indicate the economic burden of households. Appropriate coping mechanisms may be undertaken to alleviate this burden. This may have public health implications particularly in the case of households with illiterate mothers or childhood invasive enteritis, mainly in the case of girls.

## Method

### Study site

Related data were extracted from the Global Enteric Multicenter Study (GEMS), Bangladesh site database^34^. The location of the GEMS Bangladesh site was in a rural community, situated in the Mirzapur sub-district of Tangail, Bangladesh. Details about the study site have been reported elsewhere^34–36^.

### Study design and study participants

**Figure 2:**
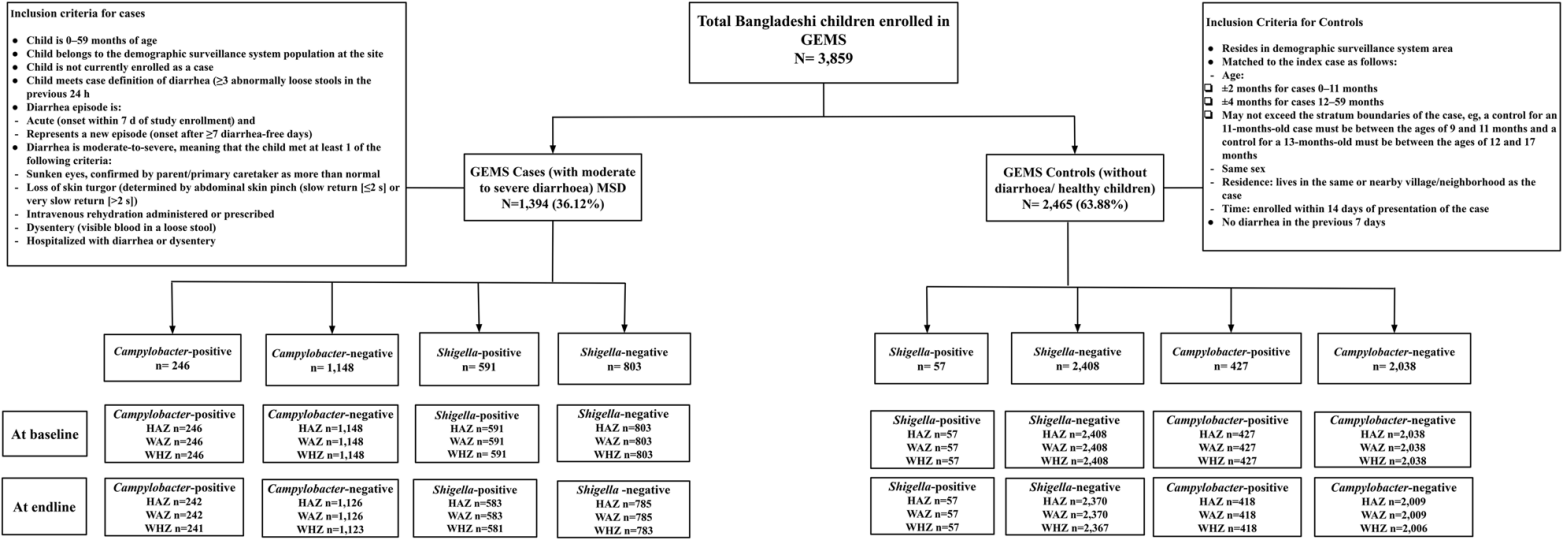
Study profile of enrolled children.

The design and methodology of the GEMS were mentioned earlier^36^. Briefly, data were extracted from cases and controls enrolled at the GEMS Bangladesh site, a three-year research during December 2007 and March 2011. GEMS was a prospective matched case–control study conducted for 36 months at 7 sites where demographic surveillance systems (DSS) regularly updated censused populations. The sampling frame comprised children aged < 60 months residing within each site’s DSS area. Children brought to sentinel health centres serving each DSS-respondent-children were assessed to match with the inclusion criteria for MSD irrespective of their socioeconomic status. Every fortnight, 8–9 cases per age stratum (0–11, 12–23, and 24–59 months) per site were targeted for enrolment. Within 14 days of each case enrolled, they undertook to enroll 1–3 randomly selected age- and sex-matched controls from the same or nearby communities^36–38^. The research had a well-defined standardized protocol for recruitment^36^. The published^37^, working hypothesis^35^, epidemiology^36^, clinical^39^, laboratory^36^, and statistical methods^40^ of GEMS have been described elsewhere^41^. In this analysis, we enrolled 1394 (36.12%) under-5 children from a total of 3859 children enrolled in the GEMS Bangladesh site. There were 648 (16.79%) *Shigella*-positive and 673 (17.44%) *Campylobacter*-positive under-5 children enrolled in Bangladesh. Among MSD cases, there were 591 (42.40%) *Shigella*-positive cases and 803 (57.60%) *Shigella*-negative controls, and 246 (17.65%) *Campylobacter*-positive cases, and 1148 (82.35) *Campylobacter*-negative controls (Fig. 2). Only 46 (< 5%) children had both *Campylobacter* and *Shigella* present in the stool.

### Collection of stool samples and stool microbiology

Stool specimens for the GEMS were examined for every child at the time of enrolment using the GEMS laboratory procedure protocol^42–44^.

### *Shigella* and *Campylobacter* spp. isolation

Tests for isolation of both *Campylobacter* and *Shigella* spp. used in GEMS have been described elsewhere^43^.

## Variable of interest

### Anthropometry

During enrollment and the 60-day follow-up visit length/ height, weight, and MUAC for each child were measured; descriptions of measurement methods are mentioned elsewhere^36,45,46^. The height/length-for-age, weight-for-age, and weight-for-height z-scores (HAZ, WAZ, and WHZ) have been calculated by WHO SAS macro using the WHO Child Growth Standards for the reference population^47, 48^.

### Diarrhea

Passage of ≥ 3 abnormally loose or watery stools per 24 h^36, 49^.

### Fever, vomiting, and dysentery

Many of the factors, such as vomiting (approximately 3 times/day) and fever on admission (measured at least 38 °C) and dysentery as the presence of visible blood in stools, can only be evaluated retrospectively^36^.

### Inclusion criteria for MSD

Every child was evaluated for diarrhea and study enrollment eligibility. The episode had to be current (initiated after around 7 days without diarrhea), acute (initiated within the previous 7 days), and at least one of the following characteristics for moderate-to-severe diarrhea (MSD) had to be met: sunken eyes (confirmed by parent or caretaker as more than usual; loss of skin turgor (abdominal skin pinch with slow [≤ 2 s] or very slow [> 2 s] recoil); intravenous rehydration administered or prescribed; dysentery (visible blood in loose stools); or hospitalized with diarrhea or dysentery^50^.

### Breastfeeding status

Breastfed referred to both exclusive and partially breastfeed children.

### Socio-demographic information

This involved data from the participant’s household (defined as a group of people sharing a cooking fire) which included mother as a primary caretaker, education of mother (illiterate or literate), and size of household (including the number of children < 5 years of age, number of people regularly sleeping in the house). The explanatory variables were known to be building materials (cement or non-cement), the practice of handwashing (before nursing or preparing baby food; after handling animals, and cleaning a child), access and the main source of drinking water (tube well and non-tube well water), water treatment (water treatment method of drinking water available or not), improved sanitation facilities (an available toilet facility for disposal of human fecal waste or not), pets on the premises (sheep, goat, rodent/fowl, cow, dog, and cat), and methods for hand washing (water with soap or without soap).

### Wealth index

Based on the wealth index quintiles (poor, lower middle, middle, upper middle, and rich), households were categorized into socio-economic status (SES) to determine potential associated factors for disease as well as indicators for constructing a wealth index for each site^36,51^.

### Duration of hospital stay

The outcome was described by using a total duration of hospital stay (less than 4 days and ≥ 4 days).

### Household follow-up visit

GEMS field staff members visited each enrolled child's household roughly 60 days after enrollment (acceptable range, 50–90 days). During these follow-up household visits, detailed comorbidity data (typhoid, pneumonia, diarrhea, and dysentery) were obtained^36^.

### Child growth

Only case–control (*Campylobacter* and *Shigella* positive and negative) sets of data on both enrollment and follow-up HAZ, WAZ, and WHZ for participants enrolled in GEMS were included in our study^36^. We used weighted means of baseline and endline HAZ, WAZ, and WAZ (n = 648 vs. 640) for *Shigella*-positive children; and HAZ, WAZ, and WAZ (n = 673 vs. 660) for *Campylobacter*-positive children irrespective of MSD from enrolment to follow-up respectively from GEMS Bangladesh site.

### Household cost

Direct medical costs, direct non-medical costs, indirect costs, and overall costs per study child (fecal *Campylobacter* and *Shigella* positive MSD) were analyzed for care-seeking from the medical facilities for the treatment of a given episode of MSD, all of which were converted to the current US dollar rate. Direct medical expenses were classified as both informal and formal expenses, with the former representing treatment given by a local healer or pharmacist and the latter combining all health centers, hospitals, and licensed practitioners. Direct non-medical costs were split down by transport and other costs, while indirect costs were either time costs or other costs.

### Statistical methods

Considering mean and standard deviation (SD) for continuous variables and frequency as a percentage to summarize the data for categorical variables, we reported the child, maternal, and household-level characteristics. Student's *t *test for continuous variables was performed to compare the mean differences, and changes in proportions were compared by the Chi-squared (*χ*^2^) test. Since *Shigella* and *Campylobacter* infection were binary indicators, we performed multiple logistic regression analyses to identify the significantly associated factors of *Shigella* and *Campylobacter* infections in children aged < 5 years for *Shigella* and *Campylobacter* positive children having MSD. The covariates were adjusted for multiple logistic regression models using a stepwise forward selection method if associated with *p *value < 0.25 in the simple model^52^, whereas other relevant variables such as age and sex were adjusted for a *p *value < 0.25 due to biological as well as public health importance as more traditional levels such as 0.05 can fail in identifying variables known to be of importance. All covariates were included in the subsequent models to obtain an adjusted final model. Adjusted odds ratios (aOR) with a 95% confidence interval (CI) as the strength of the associations were determined from multiple logistic regression. The relationship of explanatory variables (presence of fecal *Campylobacter* and *Shigella*) with the continuous outcome variables (HAZ, WAZ, and WHZ) were examined longitudinally using generalized estimating equations (GEE) with exchangeable correlation and identity link function^53,54^. The variance inflation factor (VIF) was calculated to assess multicollinearity and no variable with a VIF > 5 was identified. Some cases incurred no expenditures for both medical and total costs, and the remainder created a right-skewed distribution. For MSD cases, descriptive statistics (means and SD) for costs were estimated. The analysis of variance (ANOVA) was used to independently assess the results of the economic status of households, maternal education, sex of a child, age, and duration of illness. We determined the strength of the association by estimating the coefficients and their 95% CIs. A probability of less than 0.05 was assumed statistically significant. All data were analyzed using STATA version 15.0 IC (College Station, TX: Stata Corp LLC).

### Ethical consideration

The ethical committees and the respective research review boards at the University of Maryland School of Medicine and the committees overseeing each site and their collaborating partners from other institutions approved the clinical protocol, consent forms, case report forms, field methods, and other supportive materials prior to the start of the study. All methods were performed in accordance with the relevant guidelines and regulations. The signed informed consent for the inclusion of children in the study was obtained from the parents/guardians of the children (both sick MSD cases and healthy controls).

## Supplementary Information

Supplementary Tables.

## Data Availability

This study analyzed a publicly accessible GEMS dataset. This data can be found here: ClinEpiDB [https://clinepidb.org/ce/app/record/dataset/DS_841a9f5259].
