# Supplementary material for: Associated factors, post infection child growth, and household cost of invasive enteritis among under 5 children in Bangladesh
Source: Sci Rep. 2021 Jun 17;11:12738. doi: 10.1038/s41598-021-92132-z (PMC8211821; doi:10.1038/s41598-021-92132-z)
Supplement: Supplementary file 1 — Supplementary Tables. [file 41598_2021_92132_MOESM1_ESM.pdf]

Associated factors, post infection child growth, and household cost of invasive enteritis among under 5 children in Bangladesh

Rina Das[1], Md. Ahshanul Haque[1], Mohammod Jobayer Chisti[1], ASG Faruque[1*], Tahmeed Ahmed[1,2,3]

[1]Nutrition and Clinical Services Division, icddr,b, Dhaka 1212, Bangladesh
[2] James P. Grant School of Public Health, BRAC University, Dhaka 1212, Bangladesh
[3] Department of Global Health, University of Washington, Seattle, Washington, DC 98104, USA

* **Corresponding Author**: ASG Faruque
Emeritus Scientist
Nutrition and Clinical Services Division, icddr,b, Dhaka 1212, Bangladesh
Email: gfaruque@icddrb.org

Supplementary table S1. Copathogens isolated from the stool of *Shigella* positive and *Campylobacter* positive children having MSD

Co-pathogen isolated in stool	*Shigella*, n= 591 (%)	*Campylobacter*, n= 246 (%)
**Bacterial co-pathogen**		
Non-*Salmonella Typhi*	4	4
EAEC	121 (20.5)	67 (27.2)
ETEC	26 (4.4)	12 (4.9)
EHEC	-	-
Salmonella typhi	-	-
Vibrio cholera	3	4
Aeromonus	171 (28.9)	59 (23.98)
**Virus**		
Aedenovirus	7	5
Astrovirus	5	1
Norovirus	47 (7.9)	14 (5.7)
Rota virus	22 (3.7)	23 (9.4)
Sapovirus	10 (1.69)	2
**Eukaryote**		
Cryptosporidium	35 (5.9)	14 (5.7)
Entamoeba histolytica	43 (7.3)	18 (7.3)
Giardia	65 (11.0)	22 (8.9)

EAEC: Enteroaggregative *Escherichia coli;* ETEC: Enterotoxigenic *Escherichia coli*; EHEC: Enterohemorrhagic *Escherichia coli;* MSD; moderate-to-severe diarrhoea

Supplementary table S2. Different species isolated from fecal *Shigella* positive and *Campylobacter* positive under 5 children in Bangladesh

Species	Overall n (%)	Symptomatic (MSD) n (%)	Asymptomatic n (%)
Shigella	648 (16.79)	591 (42.40)	57 (2.31)
S. flexneri	430 (11.14)	401 (28.77)	29 (1.18)
S. sonnei	169 (4.38)	148 (10.62)	21 (0.85)
S. dysenteriae	14 (0.36)	14 (1.0)	-
S. boydii	54 (0.70)	22 (1.58)	5 (0.20)
Campylobacter	673 (17.44)	246 (17.65)	427 (17.32)
C. jejuni	508 (13.16)	194 (13.92)	314 (12.74)
C. coli	172 (4.46)	52 (3.73)	120 (4.87)

MSD; moderate-to-severe diarrhoea

Supplementary table S3. Association of fecal *Shigella and Campylobacter* with the child's HAZ, WAZ, and WHZ: results of generalized estimating equations modeling (dependent variable- HAZ, WAZ, and WHZ) [without adjusting for pathogens: *Giardia* and *Cryptosporidium*

		**Unadjusted**		**Adjusted***		**Adjusted***			
		**All**		**All**		**Asymptomatic**		**Symptomatic**	
**Pathogens**	**Z score**	**Coef. (95% CI)**	***p*-value**	**Coef. (95% CI)**	***p*-value**	**Coef. (95% CI)**	***p*-value**	**Coef. (95% CI)**	***p*-value**
***Campylobacter* (+)**	**HAZ**	0.06 (-0.03, 0.15)	0.221	0.02 (-0.07, 0.10)	0.732	-0.02 (-0.14, 0.09)	0.689	0.09 (-0.06, 0.24)	0.242
	**WAZ**	0.11 (0.02, 0.20)	0.021	0.05 (-0.04, 0.14)	0.257	-0.04 (-0.15, 0.07)	0.511	0.22 (0.06, 0.37)	**0.005**
	**WHZ**	0.10 (0.01, 0.19)	0.023	0.04 (-0.05, 0.12)	0.397	-0.07 (-0.17, 0.04)	0.220	0.22 (0.08, 0.37)	**0.003**
***Shigella* (+)**	**HAZ**	-0.11 (-0.21, -0.02)	0.015	0.001 (-0.11, 0.11)	0.972	-0.08 (-0.36, 0.19)	0.553	0.03 (0.09, 0.16)	0.640
	**WAZ**	-0.31 (-0.40, -0.21)	<0.001	-0.06 (-0.17, 0.05)	0.282	-0.04 (-0.32, 0.24)	0.766	-0.04 (-0.17, 0.09)	0.512
	**WHZ**	-0.41 (-0.50, -0.32)	<0.001	-0.11 (-0.21, -0.001)	**0.046**	-0.01 (-0.28, 0.26)	0.952	-0.10 (-0.23, 0.02)	0.110

***** adjusted for age, gender, MSD, breastfeeding status, mother's education, number of people regularly sleep in the house, number of under 5 children at house, hand washing before nursing a child and after cleaning the child, handwashing material, main source of drinking water, available toilet facility, wealth index, and comorbidity (malaria, typhoid, pneumonia, diarrhea, dysentery)

OR: odds ratio, CI: confidence interval

Supplementary table S4. Mean household costs (U.S. dollars) by type of household cost associated with *Campylobacter* and *Shigella* diarrheal illness in Bangladesh

	Shigella (+)			***Campylobacter (+)***		
**Cost**	**N**	**Mean cost**	**SD***	**n**	**Mean cost**	**SD***
**Cost by type**		USD			USD	
Direct medical	591	2.95	2.04	246	2.32	1.01
Direct nonmedical	44	0.34	0.38	9	0.37	0.36
Total direct	591	2.97	2.06	246	2.34	1.04
Indirect cost	220	3.22	2.51	60	4.74	6.17
Total cost	590	4.17	3.64	246	3.49	4.14

*SD: Standard Deviation; USD: US Dollar
